# Suicide among Cancer Patients: Current Knowledge and Directions for Observational Research

**DOI:** 10.3390/jcm12206563

**Published:** 2023-10-16

**Authors:** Ben Grobman, Arian Mansur, Dolapo Babalola, Anirudh P. Srinivasan, Jose Marco Antonio, Christine Y. Lu

**Affiliations:** 1 Harvard Medical School, Boston, MA 02115, USA; bgrobman@hms.harvard.edu (B.G.); arianmansur@hms.harvard.edu (A.M.); 2 College of Medicine, University of Ibadan, Ibadan 200285, Nigeria; dbabalola302@stu.ui.edu.ng; 3 Harvard College, Cambridge, MA 02138, USA; anisrinivasan@college.harvard.edu (A.P.S.); marckyantonio@college.harvard.edu (J.M.A.); 4 Harvard Pilgrim Health Care Institute, Harvard Medical School, Boston, MA 02215, USA; 5 Faculty of Medicine and Health, School of Pharmacy, The University of Sydney, Sydney, NSW 2050, Australia; 6 Kolling Institute, Faculty of Medicine and Health, The University of Sydney, The Northern Sydney Local Health District, Sydney, NSW 2064, Australia

**Keywords:** cancer, suicide, methodological review, health policy, observational studies

## Abstract

Cancer is a major public health concern associated with an increased risk of psychosocial distress and suicide. The reasons for this increased risk are still being characterized. The purpose of this study is to highlight existing observational studies on cancer-related suicides in the United States and identify gaps for future research. This work helps inform clinical and policy decision-making on suicide prevention interventions and ongoing research on the detection and quantification of suicide risk among cancer patients. We identified 73 peer-reviewed studies (2010–2022) that examined the intersection of cancer and suicide using searches of PubMed and Embase. Overall, the reviewed studies showed that cancer patients have an elevated risk of suicide when compared to the general population. In general, the risk was higher among White, male, and older cancer patients, as well as among patients living in rural areas and with lower socioeconomic status. Future studies should further investigate the psychosocial aspects of receiving a diagnosis of cancer on patients’ mental health as well as the impact of new treatments and their availability on suicide risk and disparities among cancer patients to better inform policies.

## 1. Introduction

In 2023 alone, 1,958,310 new cancer cases are expected to be diagnosed in the United States, with approximately one-third of that number estimated to die from their illness [1]. Despite numerous medical advances and increasing patient survival, cancer remains the second leading cause of death in the United States [2]. Cancer is thus a major public health concern, and this devastating diagnosis is exacerbated by concomitant psychological, emotional, and financial strain [3]. The difficult trajectory of cancer treatment, recovery, and the possibility of death from disease puts patients at a high risk of psychological distress and suicide [4]. While cancer is often the primary cause of death in patients diagnosed with cancer, studies have shown that cancer patients have an increased risk of depression and anxiety [5] and of suicide death when compared to the general population with up to 11 times more estimated risk [6,7]. Previous research has established that complex socioeconomic and demographic factors influence suicide risk in cancer patients [6]. However, because of the multifactorial nature of mental health, there is not a clear understanding of the interplay between these factors in determining suicide risk in cancer patients. In particular, there have been significant advances in cancer treatment over the last two decades that have extended patients’ lifespans. Observational studies are valuable in examining these intersections because of their longitudinal and wide capture of influencing factors such as social determinants of health, reliable death data, and large sample sizes. These characteristics can account for the relatively low occurrence, but nonetheless devastating consequence, of suicide among cancer survivors. As such, analyzing observational studies provides an effective way of understanding this intersection regarding demographic, geographic, and chronologic differences in the suicide rate among cancer patients and, importantly, the trends of such interactions over time.

In this review, we highlight existing observational studies on cancer-related suicides in the United States with the aim of describing current knowledge as well as highlighting gaps in the literature. We anticipate that this summary will be useful to inform clinical and policy decision-making on suicide prevention interventions, and future research on the detection and quantification of suicide risk among cancer patients. 

## 2. Methods

### 2.1. Search Strategy

**Figure 1:**
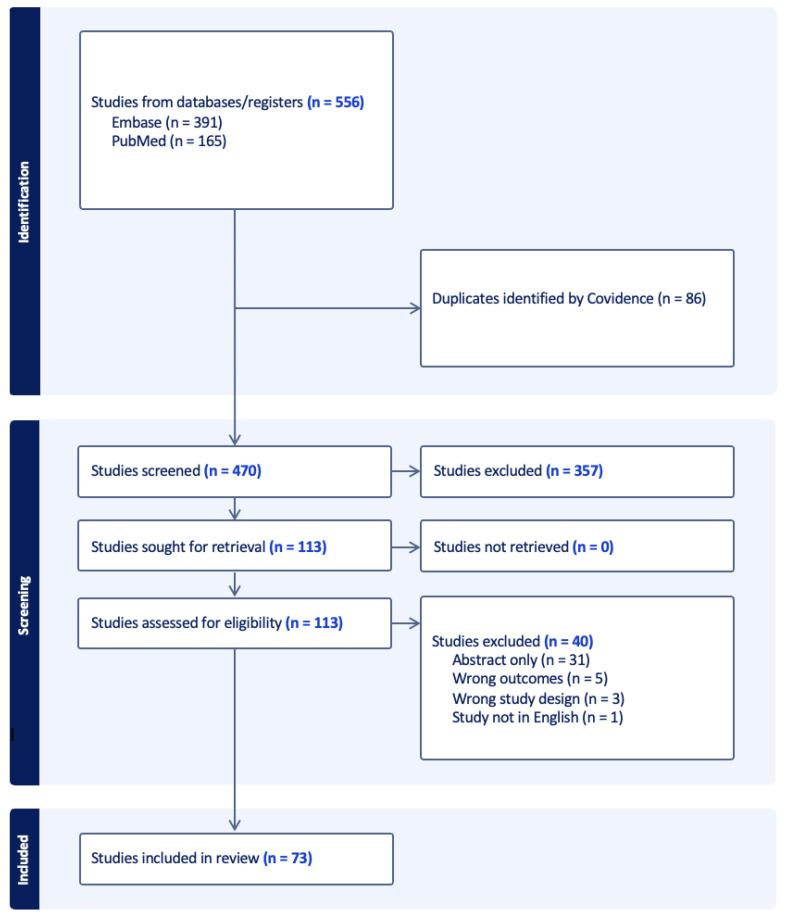
Preferred Reporting Items for Systematic Reviews and Meta-Analyses (PRISMA) flow diagram.

We sought to identify peer-reviewed, original investigations that used observational databases to assess the risk of suicide deaths among people with cancer in the United States. We searched for articles using PubMed and Embase in October of 2022. In PubMed, we especially searched for “Cancer” AND “Suicide” AND (“database” OR “SEER” OR “wonder”) because the Surveillance, Epidemiology, and End Results (SEER) and the Centers for Disease Control and Prevention’s Wide-ranging Online Data for Epidemiologic Research (CDC WONDER) are two large, reliable data sources for cancer and mortality-related analyses (including suicide death). In Embase, we used MeSH terms and searched for (‘cancer’/exp OR ‘cancer’) AND (‘suicide’/exp OR ‘suicide’) AND (‘database’/exp OR ‘database’ OR ‘cancer registry’/exp OR ‘cancer registry’) AND (‘united states’/exp OR ‘united states’). We were particularly interested in recent developments in the literature and wanted to account for the recent advancements that have prolonged lifespan among cancer patients, so we limited our search to studies published after 2010. Our search obtained 391 results from Embase and 165 results from PubMed. Covidence was used to remove duplicates. Review articles, studies that did not use observational methods, studies not in English, and studies that were not full articles were excluded. We excluded studies that were not full articles as we were interested in understanding the subtleties in the methodological nature that these studies employed as well as the results to better identify research gaps and elucidate the interactions between various variables and suicide risk. Additionally, conference abstracts may have lower scientific validity due to often preliminary results and a less rigorous peer-review process. Titles and abstracts were screened independently by two authors (B.G. and A.P.S.). Full-text articles were screened independently by three authors (B.G., M.A., and A.M.) (Figure 1).

### 2.2. Data Extraction

We manually extracted data on each study’s characteristics. Characteristics extracted were each study’s primary objective, the database used, the time period analyzed, the primary study population, the populations to which rates of suicide were compared, the study design (e.g., cross-sectional vs. longitudinal), the geographic regions included, the covariates (e.g., sex, age, race, and geography) included, and the statistical analyses performed (e.g., standardized mortality ratios). For each study, we also briefly summarized the study’s main findings. 

## 3. Results

### Search Results

A total of 556 records were identified from searches in PubMed (n = 165) and Embase (n = 391). After removing 86 duplicates and applying our inclusion/exclusion criteria, 73 articles were selected for review (Table 1). Table 1 summarizes key data extracted on each study’s characteristics. Table S1 summarizes the breakdown of key features, such as database used, study design, regions included, covariates analyzed, statistical analysis, and cancer types examined across the studies. Of the 73 studies, a majority (n = 69, 95%) used the Surveillance, Epidemiology, and End Results (SEER) Program database. 

With respect to the cancer types studied, 20 (27%) studies analyzed all cancer types. Other studies focused on specific cancer types, of which genitourinary cancers were the most common in 13 (18%) studies. This was followed by nine studies examining head and neck cancer (12%), five studies examining gastrointestinal cancer (7%) and three studies each which examined lung and bronchus cancers, breast, and gynecological cancers (4%). Most studies analyzed sex (n = 60, 82%), age (n = 58, 79%), and race (n = 69, 95%). Fifty-three studies (73%) studies analyzed time as a covariate; of these studies, 46 (63%) included the calendar year as a covariate, and 29 (40%) included the time since diagnosis. By contrast, only 18 (25%) studies analyzed geography (e.g., rural vs. urban) as a covariate and 20 (27%) studies examined socioeconomic-related variables.

In studies that analyzed all cancer types (n = 20), estimates for the overall risk increase of suicide among cancer patients ranged from 1.41–4.44 [4,8]. Particularly high risks were found for pancreatic cancer (SMR = 6.43, 95% CI: 5.49–7.37) and esophageal cancer (SMR = 5.45, 95% CI: 4.66–6.35) [9,10]. By contrast, no effect was seen for prostate cancer (SMR = 0.95, 95% CI: 0.90–1.00) and a reduction in risk was seen for thyroid cancer (SMR = 0.59, 95% CI: 0.50–0.69) [11,12]. 

Thirteen studies examined the risk of suicide among patients with genitourinary cancers (overall SMR range 0.95–2.71) [12,13]. Multiple studies found that patients with genitourinary cancers were at a generally increased risk of suicide. However, this risk increase varied based on the exact type of cancer, cancer severity, and the treatments received [13,14,15,16]. A 2015 paper by Klaassen, et al. used SEER data from 1988 to 2010 and found that, as compared with the age-matched US population, patients with bladder (SMR—2.71, 95% CI: 2.02–3.62) and kidney cancer (SMR = 1.86, 95% CI: 1.32–2.62) were at a high risk of suicide, patients with prostate cancer had the highest risk of suicide with increasing time from diagnosis (SMR = 1.84, 95% CI: 1.39–2.41), and patients with bladder cancer had the highest risk of suicide within the first 5 years after diagnosis (SMR = 3.05, 95% CI: 2.26–3.96) [16]. A 2016 paper by Klaassen, et al. that examined SEER data from 1988 to 2010 also found that among patients with bladder cancer, radical cystectomy further increased patients’ suicide risk as compared to the general population (SMR = 3.54, 95% CI: 2.70–4.54) [13]. A 2022 paper by Yu, et al. examined SEER data from 2004 to 2015 and found that among patients with renal cell carcinoma, patients at most stages did not have an elevated risk of suicide, but patients with stage IV cancer had a significantly elevated risk of suicide as compared to the general population (SMR = 2.24, 95% CI: 2.11–2.38) [15]. 

Nine studies investigated suicide risk among patients with head and neck cancer with a risk increase ranging from 1.97–3.21 [17,18]. Massa, et al. examined SEER data from 2004 to 2011 and found that patients with head and neck squamous cell carcinoma had a risk of competing causes of death 11.3 times higher than people in the general population [19]. A 2018 paper by Osazuwa-Peters, et al. examined SEER data from 2000 to 2014 and found that survivors of head and neck cancer had a significantly elevated risk of suicide, even when compared to patients with cancers of other common sites (SMR = 3.21, 95% CI: 2.18–4.23) [20]. Kam, et al. examined SEER data from 1973 to 2011 and found that while patients with head and neck cancer in general were at an elevated risk of suicide (ARR = 1.97, 95% CI: 1.77–2.19), this effect was most pronounced among patients with hypopharyngeal (SMR = 13.91, 95% CI: 11.78–16.03) and laryngeal (SMR = 5.48, 95% CI: 4.14–6.81) cancers [18]. 

Five studies examined patients with gastrointestinal cancers with SMRs ranging from 3.21–5.45 [10,18]. Chen, et al. examined SEER data from 1975 to 2016 and found that esophageal cancer patients were at five and a half-fold risk of suicide, and this risk was particularly among those who were older, male, and who did not receive surgery or chemotherapy [10]. While studies consistently showed that patients with gastric cancer were at an elevated risk of suicide, subgroup analyses provided more heterogeneous results. Bowden, et al. used SEER data from 1973 to 2013 and found that female gender, White race, age ≤ 39 years, and age 70–79 were associated with an increased risk of suicide among gastric cancer patients [21]. However, Sugawara, et al. used SEER data from 1998 to 2011 and found that suicide rates were highest within 3 months of a gastric cancer diagnosis and that male sex, White race, and unmarried status were associated with an increased risk of suicide [22]. Similarly, Elshanbary, et al. used SEER data from 1975 to 2016 and found that while patients with gastric adenocarcinoma had a higher risk of suicide, the risk was lower within this population among Black and female patients [23]. 

Three studies examined patients with cancers of the lung and bronchus; one paper reported an overall SMR of 4.95 (95% CI: 4.68–5.24). Zhou, et al. used SEER data from 1973 to 2013 and found that male, unmarried, and elderly patients with non-small cell lung cancer were at a particularly increased risk of suicide mortality [24]. Urban, et al. used SEER data from 1973 to 2009 and found that while all patients with lung cancer have an increased risk of suicide immediately after diagnosis, the risk was particularly elevated among those with a poorer prognosis such as those with metastatic disease (SMR = 9.52) [25]. 

Three studies examined patients with breast cancer. Gaitanadis, et al. used SEER data from 1973 to 2013 and found that younger age, single marital status, and receipt of cancer surgery were predictors of committing suicide [26]. 

Three studies examined outcomes among patients with gynecological cancers. Ward, et al. used SEER data from 1973 to 2007 and found that women with gynecologic malignancies were more likely to commit suicide than women with non-gynecologic malignancies (RR = 1.3, 95% CI: 1.1–1.5) [27]. The study by Mahdi, et al. used SEER data from 1988 to 2007 and found that among women with gynecologic malignancies, a younger age at diagnosis, high-grade disease, and the absence of surgical intervention were associated with higher suicide risk [28]. 

Only three studies examined longitudinal trends in suicide rates [29,30,31]. While Yu, et al.’s study showed that suicide rates have been on the rise over the past three decades of the study’s time, the study was limited to patients with oral cavity and oropharyngeal cancer and had older years of data up to 2007 [30]. The study conducted by Rahouma, et al. analyzed standardized mortality ratios (SMRs) of breast, prostate, colorectal, and lung cancers over time but only has data up to 2013 [31]. The study conducted by Han, et al., which analyzed CDC WONDER data, was the only longitudinal trend study found in our search that analyzed all cancer types with more recent years of data up to 2018 [29]. They found that cancer-related suicide rates have been declining over the past two decades while suicide rates among the general population have been rising, which may be attributed to the increasing role of psycho-oncology, palliative, and hospice care in cancer patients [29].

Studies on the intersection of cancer and suicide have consistently shown that male cancer patients are more prone to suicide than female cancer patients [32,33,34]. Yang, et al. found that two-thirds of suicide deaths among patients with leukemia happened among males [32]. Zhou, et al. found that male patients with intracranial tumors had a significantly higher rate of suicide death and that 83% of suicides among patients with intracranial tumors were among males [33]. Kendal and Kendal similarly found that male gender increased the risk of suicide among cancer patients by a factor of six, and that 82.8% of cancer patients who committed suicide were male [34]. 

Regardless of the type of cancer, race has been identified to be a determinant of suicide risk among cancer patients. Several studies have shown that White cancer patients have the highest suicide risk in the United States population [16,21,35,36]. Bowden, et al. found that White race was associated with a four-fold increase in the risk of suicide among patients with gastric cancer as compared to the general population [21]. Yu. et. al, found that White race was associated with a significantly elevated risk of suicide among leukemia patients when compared to Black patients (HR = 6.80, 95% CI = 1.69–27.40, *p* = 0.007) [35]. Ma, et al. found that 92.4% of patients with a primary solid tumor who committed suicide were White [36]. Klaassen, et al. found that Black patients with bladder cancer had a significantly lower risk of suicide as compared to White patients (OR range 0.26–0.46) [16].

Multiple studies showed that older cancer patients had higher rates of suicide than younger cancer patients [16,33,35,37]. Zhou, et al. showed that compared to patients aged ≤39 years, patients aged 60–79 with intracranial tumors had an elevated risk of suicide (HR = 2.28, 95% CI: 1.35–3.86) [33]. Similarly, Chen, et al. found that among patients with hepatocellular carcinoma, older patients had a higher risk of suicide when compared to younger ones. (HR = 2.28, 95% CI: 1.21–4.31) [37]. An exception to this pattern was seen in a study on suicide mortality among breast cancer patients by Gaitanidis, et al. which showed that compared to patients 70 or older, patients aged 30 or younger and those aged 30–49 had a higher risk of suicide when compared to those aged <30 [26]. 

Multiple studies showed that cancer patients who are unmarried are more likely to commit suicide than married cancer patients [22,38]. Sugawara and Kunieda found that among patients with gastric cancer, unmarried patients were more likely to commit suicide than married patients [18]. Du, et al. found that, as compared to married cancer patients, single (HR = 1.76, 95% CI: 1.55–2.00, *p* < 0.001) and divorced patients (HR = 2.20, 95% CI: 1.89–2.56, *p* < 0.001) were at a higher risk of suicide [38]. 

Regarding geography, Su, et al. showed that cancer patients living in urban areas were at a lower risk of suicide as compared to their rural counterparts [39]. Suk, et al. similarly found that while patients diagnosed with cancer were at an elevated risk of suicide regardless of their urban or rural status, cancer patients living in rural areas had a larger increase in their risk of suicide than patients living in urban areas [8]. Abdel-Rahman similarly found that in counties with <50% of residents living in urban areas, the increase in suicide rate among cancer patients was higher than in counties where >50% of residents lived in urban areas [40]. Osazuwa-Peters, et al. also found that compared with head and neck cancer patients living in rural areas, people with head and neck cancer living in urban and metropolitan areas had a lower suicide risk [20]. 

Multiple studies also found a significant effect of socioeconomic status on the rate of suicide among cancer patients. Yang, et al. found that a higher median household income was associated with a lower risk of suicide among patients with leukemia [32]. Abdel-Rahman found that across multiple measures of county-level socioeconomic status, lower socioeconomic status was associated with a higher risk of suicide among cancer patients [40]. In counties where ≥20% of residents had less than a high school education, there was a larger elevation in suicide risk among cancer patients as compared to counties where <20% of residents had less than a high school education. In counties where >5% of the population lived below the poverty line, there was an elevation in suicide risk among cancer patients as compared to the general population. This study also found that in counties with an unemployment rate <5% there was a smaller elevation in suicide risk than in counties with an unemployment rate >5%. By contrast, Walsh, et al. found that among thyroid cancer patients, household income had no effect on suicide risk [11].

## 4. Discussion

In this review, we summarized the observational studies published since 2010 that examine the risk of suicide among cancer patients. Despite advances in cancer treatment over the last two decades that have extended patients’ lifespans, a large proportion of the studies in our review showed that cancer patients are at an elevated risk of suicide as compared to the general population. The risk was higher among White cancer patients, male cancer patients, older cancer patients, and among patients living in rural areas.

The finding that male cancer patients are more prone to suicide than female cancer patients aligns with findings from the general population, which show that males have higher rates of death from suicide, despite females having higher rates of suicidal ideation and more suicide attempts [41]. While the reasons for this disparity are multifactorial, previous explanations have included a higher lethality of the methods used for suicide, lower treatment rates for depression, and higher rates of alcohol misuse among men as contributing factors [41,42]. 

Our findings regarding racial disparities in suicide among cancer patients are similar to studies from the general population which show that there are higher rates of suicide among White Americans as compared to Black Americans [43]. Notably, this disparity persists even when examining suicide rates among Black and White Americans living in the same city, indicating that this disparity exists regardless of geography. While the reasons for this disparity are incompletely known, previous studies have attributed this difference to a higher prevalence of religious involvement among Black Americans [44]. Another proposed reason for this disparity is higher rates of misclassification of suicides as being due to causes other than suicide among Black Americans [45]. This is in part because Black suicide decedents are less likely to carry psychiatric diagnoses on their death certificates, and medicolegal personnel in charge of determining cause of death may be less likely to attribute deaths to suicide when the decedent does not carry a psychiatric diagnosis [46]. Recent studies have shown that suicide is rising among Black Americans, whereas the suicide rate has recently decreased among White Americans [47]. While the reasons for these changing patterns are incompletely elucidated, previous studies have attributed these patterns to economic changes throughout the first two decades of the century as well as the continued effects of racism on minorities in the United States [48]. However, given the current lack of knowledge, future studies on the intersection of racial disparities and suicide among cancer patients should examine whether these changing patterns in the overall population manifest themselves among cancer patients, and should further examine the underlying reasons for these changing patterns. 

Our findings regarding age are similar to those of the general population, in which older age is associated with a higher risk of suicide. Possible reasons for this association may be a lower quality of life among elderly patients, worse mental health, and a higher degree of acceptance of death [35]. Additionally, elderly patients may be more likely to experience social isolation, increased rates of chronic disease, and a loss of independence [49]. Social isolation and loneliness may also explain the higher suicide risk among unmarried cancer patients [50]. 

The studies examined in this paper found that marriage was a protective factor against suicide. Some prior studies examining the general population have also described marriage as a protective factor for suicide, possibly due to improved social support and financial status [51]. However, while studies have found this relationship to be consistent among men, the association between marital status and suicide among women is less clear, and some studies have found no association between marriage and suicide among women [52,53]. This effect may be because, on average, women take on a disproportionate burden of child-rearing and the provision of social support within marriages, leading to benefits for the male partner that they themselves do not experience [52]. However, another study from Norway found that marriage is a protective factor against suicide for both men and women, possibly due to societal differences in the way that marriages are structured [54]. Future studies should further investigate the relationship between marital status and suicidality among cancer patients, particularly with regard to issues of gender and societal divisions of domestic labor. 

Our finding that cancer patients living in rural areas were at a higher risk of suicide than their urban counterparts aligns with findings from previous studies in the general population. A recent meta-analysis showed that across high-income English-speaking countries, rurality is associated with a higher risk of suicide. However, a subgroup analysis showed that this relationship was only significant for males, while females living in rural areas did not have a higher risk of suicide than their urban counterparts [41,42,43,55]. While the reasons for this disparity are incompletely established, possible explanations include decreased social support and decreased access to necessary mental health services [56].

Most studies showed that cancer patients with lower socioeconomic status were at a higher risk of suicide. This relationship is consistent with findings in the general population across multiple countries. For example, a 2017 study from Korea showed that people with lower household income were at a higher risk of suicide, and that this rate increased across income deciles, so that people in the lowest income decile had more than three times the suicide risk of people in the highest income decile [57]. A 2023 study from the United States found that higher county-level social vulnerability (measured by the Social Vulnerability Metric which incorporates socioeconomic factors such as county-level average unemployment, education, insurance, and housing) was associated with a higher suicide rate [58]. 

Importantly, this investigation has identified several major gaps in knowledge. First, certain cancer types have been shown to result in an increased risk of suicide in patients compared to other forms of cancer; however, why this occurs is still uncertain. Multiple studies in our analysis showed that cancer patients are at a dramatically increased risk of suicide in the first few months after receiving a diagnosis [9,22,59,60]. Even as treatments and resources have improved, the effect of diagnosis itself on suicide risk requires further exploration, particularly with regard to how this effect may vary by socioeconomic status. While cancer treatments have developed rapidly over the past several decades and patient survival rates have significantly improved, very few studies have tracked suicide rates among cancer patients over time. Disparities in access to new cancer treatments by race/ethnicity, socioeconomic status, and geography are well-documented [61,62,63] but how these might interact with suicide rates is still poorly understood. Longitudinal observational studies are required to examine suicide risk across cancer types, race/ethnicity, and socioeconomic status groups, and by geography. Such studies can inform if disparities are exacerbating or improving over time as cancer care and suicide prevention evolve and can identify where further efforts might be needed to target certain vulnerable subgroups.

### 4.1. Clinical Implications

This study has important clinical implications, given that cancer is a highly prevalent condition associated with significant morbidity and mortality. Understanding the risk of suicide among patients diagnosed with cancer with regard to cancer site, time, and sociodemographic factors is essential in properly tailoring suicide prevention screening efforts and psycho-oncological treatment to these patients. Our study is one of the first to comprehensively review the various studies which have addressed these interactions. It is crucial for clinicians to understand which cancer patients may be at the highest risk of suffering serious psychological consequences, as suicide is preventable. 

### 4.2. Study Limitations

This study has several limitations. First, the employed search strategy may have missed articles despite efforts to collect all published studies pertaining to the effect of cancer on patient suicide risk. We only used two databases in our search. While these two are well-established, other ones like Web of Science and Cochrane may have identified others and should be considered in future research. Second, the methodological quality of the studies used were not assessed prior to analysis. Third, our report did not summarize suicide risk due to differences across studies such as cancer type, time periods analyzed, and changing covariates. Finally, there are inherent limitations with observational studies, such as residual confounding and inadequate capture of patients’ clinical histories, which may be relevant to understand the determinants of suicide risk; these impact our ability to fully elucidate the reasons for the increased risk of suicide among patients with cancer. Future studies should address the gaps discussed, particularly the effect of diagnosis on patient suicide risk and the relationship between quality of care and cancer patients’ mental health [64,65]. 

## 5. Conclusions

In conclusion, cancer patients have an elevated suicide risk compared to the general population, and this elevated risk is especially seen among White, male, and older cancer patients and those living in rural regions and with low socioeconomic status. Further understanding of why this elevated risk exists is critical to mitigating this key preventable healthcare issue and informing new policies. Future studies should further investigate the psychosocial aspects of receiving a diagnosis of cancer on patients’ mental health as well as the impact of new treatments and their availability on suicide risk and disparities among cancer patients to better inform policies.

## Figures and Tables

**Table 1:** Aggregated summary statistics.

	N	%
Studies included	73	100
PubMed indexed	71	97
Database used SEER	69	95
Other	5	7
Comparison group		
US general population	47	64
Other	14	19
Study design		
Cross-sectional	70	96
Longitudinal data	53	73
Longitudinal trends	3	4
Regions included		
Full US	72	99
California	1	1
Covariates analyzed Sex	60	82
Age	58	79
Race	69	95
Time	51	70
Geography	18	25
Socioeconomic status	20	27
Cancer types analyzed		
All	20	27
Genitourinary	13	18
Head and neck	9	12
Gastrointestinal	5	7
Lung and bronchus	3	4
Breast	3	4
Gynecological	3	4
All solid	2	3
Hematologic	2	3
Brain	2	3
Pancreas	2	3
Bone and soft tissue	2	3
Skin	1	1
Other	3	4

Abbreviations: SEER—Surveillance, Epidemiology, and End Results; US—United States.

## Data Availability

Not applicable.

## Supplementary Materials

The following supporting information can be downloaded at: https://www.mdpi.com/article/10.3390/jcm12206563/s1, Table S1: Summary of Observational Studies on Cancer and Suicide. References [66,67,68,69,70,71,72,73,74,75,76,77,78,79,80,81,82,83,84,85,86,87,88,89,90,91,92,93,94,95,96,97,98] cited in the Supplementary Materials.
