# Supplementary material for: Suicide among Cancer Patients: Current Knowledge and Directions for Observational Research
Source: J Clin Med. 2023 Oct 16;12(20):6563. doi: 10.3390/jcm12206563 (PMC10607431; doi:10.3390/jcm12206563)
Supplement: Supplementary file 1 [file jcm-12-06563-s001.zip › jcm-2623578-supplementary.pdf]

Journal of
Clinical Medicine

MDPI

Table S1. Summary of Observational Studies on Cancer and Suicide.

Last Name of First Author	Publication year	Aims/Objectives	Database Used	Years Analyzed	Study population	Comparisons	Geographic Regions Included	Stratifications/ Covariates	Outcome Measures	Quantitative Outcomes	Main Results
Nasseri [66]	2012	Calculate and compare the age-adjusted rates and standardized mortality ratios for suicide in cancer patients by sex and race/ethnicity, and to use age-adjusted rates to describe suicide patterns within cancer patient population by sex, organ site, stage at diagnosis, and marital status.	California Cancer Registry (part of SEER)	1997-2006	Patients diagnosed with cancer	California general population	California	Sex; Race; Other: Cancer site, marital status, cancer stage	Age-adjusted rates, SMR	Rate Ratio = 2.5, SMR = 2.0	Cancer patients are at higher risk of suicide than the general population
Komic [67]	2017	Assess the incidence of suicide among patients with melanoma of the skin, and to identify demographic, clinical, and pathologic factors associated with suicide in this patient population.	SEER	1988-2013	Patients with melanoma of the skin	US general population	United States	Sex; Age; Race	OR, SMR	Overall not reported	Patients with malignant melanoma were found to have a statistically higher risk of suicide compared with the general population

Walsh [11]	2018	Examine suicide incidence and associated factors in thyroid cancer patients	SEER	1973-2013	Patients with thyroid cancers	US general population	United States	Sex; Age; Race; Time; Other: Income, marital status, stage at diagnosis, primary site, treatment received, tumor histopathology	SMR, OR	Overall SMR = 0.59, 95% CI: 0.50-0.69	Female and White patients with thyroid cancer are more likely to commit suicide than the demographically matched general US population.
Choi [68]	2019	Examine (1) how decedents with physical health problems recorded as a suicide precipitant differ from those whose health problems were not recorded as a precipitant on depressed mood and other sociodemographic factors and suicide means, and (2) frequently mentioned physical health problems and/or related concerns in coroner/medical examiners and law enforcement reports	NVDRS	2005-2014	All suicide decedents between 2005 and 2014, specifically focused towards those aged 65 or older	N/A	United States	Sex; Age; Race; Time; Other: Precipitating factors, suicide method	Adjusted OR with 95% CI	Overall not reported	One-half of suicide decedents aged 65 or older (and 60% among decedents aged 85+) had physical health problems recorded as having directly contributed to their suicide. Those older adults were more likely to have been suffering from depression and to have expressed their suicidal intent than those without physical health problems recorded as a precipitant.

Yang [32]	2020	Analyze sociological risk factors for suicide death in leukemia patients	SEER	1973-2015	Leukemia patients older than 18 years	N/A	United States	Sex; Age; Race; Time; Geography; Other: Marital status at diagnosis, poverty level, educational status, unemployment status, income, smoking status	OR, univariate and multivariate logistic regression analysis	Overall not reported	Younger age at diagnosis, being male, having at least a bachelor's degree, and being from California significantly increased the risk of suicide death.
Shivarov [69]	2022	Assess whether cancer diagnosis interacts with the seasonality of suicides	SEER	1973-2016	Patients with primary malignancies and documented cause of death Suicide reported in SEER	N/A	United States	Sex; Age; Race; Time; Geography; Other: Main cancer subtypes	Cosinor model	P value cosine term = 1.68×10^{-7} P value sine term = 3.63×10^{-10}	There was a significant peak in suicides among cancer patients in May. This aligns with what is found in the general US population.
Beard [70]	2013	Quantify non-testicular cancer causes of death and associated temporal patterns among a large number of men with stage I seminoma in a population-based setting	SEER	1973-2001	Men diagnosed with stage I testicular seminoma	2000 US population	United States	Age; Race; Other: Tumor stage, county education level, receipt of adjuvant radiotherapy	Overall mortality and mortality from second malignant neoplasm, cardiovascular disease, infection, suicide, cerebrovascular disease, COPD	SMR = 1.45, 95% CI: 1.06- 1.98	Among patients with stage I testicular seminoma, excess cardiovascular deaths were not observed, but there was excess mortality from second malignant neoplasms, infection, and suicide.

Ward [27]	2013	Describe the risk of suicide among women with invasive gynecologic malignancies compared to women diagnosed with non-gynecologic malignancies	SEER	1973-2007	Women with gynecologic malignancies	Women with non-gynecologic malignancies	United States	Race; Other: Marital status	Relative risk ratio for suicide	RR = 1.3, 95% CI: 1.1 – 1.5	Women with gynecologic malignancies were 30% more likely to commit suicide than were women with non-gynecologic malignancies
Yu [15]	2022	Explore the mortality patterns of contemporary renal cell carcinoma survivors	SEER	2004-2015	Patients diagnosed with renal cell carcinoma	US general population	United States	Sex; Age; Race; Time; Other: Cancer stage, histology, treatment received, marital status	SMR	Stage IV SMR = 3.46, 95% CI: 2.17-5.24	Non-renal cell carcinoma causes of death account for more than 3/4 of deaths among renal cell carcinoma patients with stage I/II disease. Stage IV patients had an elevated risk of suicide.
Klaassen [13]	2016	Evaluate the suicide incidence in patients with bladder cancer and assess the impact of radical cystectomy on suicide rates.	SEER	1988-2010	Patients diagnosed with bladder urothelial carcinoma	US general population	United States	Sex; Age; Race; Other: Marital status, tumor stage, treatment received	SMR	SMR = 2.71, 95% CI: 2.02–3.62	A diagnosis of bladder cancer increases the incidence of suicide compared to the general population
Klaassen [14]	2018	Identify specific factors, temporal trends and potential geographic differences in patients with bladder cancer. Identify patients at high risk for late suicide after diagnosis.	SEER	1973-2013	Patients with bladder urothelial carcinoma	N/A	United States	Sex; Age; Race; Time; Geography; Other: Treatment received, marital status, cancer stage	HR for suicide, OR for suicide	Overall not reported	Early year of diagnosis, unmarried status, white race, elderly age, male sex, regional stage, living in the Southeast, Wester, and Midwest US, and not undergoing radical cystectomy are associated with suicidal death among bladder cancer patients.

Liu [60]	2022	Estimate the suicide risk of patients with cancer, as compared with that of the general US population, depending on various sociodemographic factors and cancer types	SEER	1975-2016	Patients with cancer	US general population	United States	Sex; Age; Race; Time; Other: Tumor stage, Marital status, Insurance status	Absolute excess risk estimates, SMR	SMR = 1.45, 95% CI: 1.42 – 1.49	Cancer patients in the US experienced a 45% increase in suicide risk when compared with the general population.
Massa [19]	2017	Describe the competing causes of mortality in the head and neck squamous cell carcinoma (HNSCC) population, compare mortality rates to the general population, and identify risk factors associated with death from these competing causes.	SEER	2004-2011	Patients with primary HNSCC (oral cavity, oropharynx, nasopharynx, hypopharynx, larynx, sinonasal)	US general population	United States	Sex; Age; Race; Other: tumor site, tumor stage	Cause of death frequencies, SMR, competing mortality risk regression	Overall not reported	HNSCC patients in this study had death rates 11.3 times higher than the general population for competing causes. Chronic obstructive pulmonary disease and cardiovascular disease have high SMRs and also constitute a substantial proportion of deaths. Mortality from chronic liver disease and suicide were particularly elevated, approaching 40 times the rates in the general population.
Johnson [59]	2012	Characterize suicide risk within the first year of cancer diagnosis to potentially identify a clinically useful window of peak suicide risk	SEER	1973-2005	Individuals at least 20 years old diagnosed with cancer	N/A	United States	Sex; Age; Race; Time; Other: Tumor grade, tumor stage, cause of death	Frequency analysis, one-way ANOVA	33.2% of suicides occurred within 1 month	Cancer patients have an increased risk of suicide. This risk peaks with the month following diagnosis.

Zhou [33]	2022	Investigate the incidence, trend and risk factors associated with suicide among patients with malignant intracranial tumors	SEER	1975-2015	Patients diagnosed between 1975 and 2016 with malignant intracranial tumors	US general population	United States	Sex; Age; Race; Time; Other: Marital status, Urbanization, Socioeconomic status, Pathology, tumor site, first malignancy, treatment received	SMR	1.90 (95% CI: 1.55 – 2.30)	The suicide mortality among patients with malignant intracranial tumors steadily elevated in the past decades. Male sex, older age, and supratentorial location were significantly associated with risk of suicide, especially within the first year following diagnosis.
Aboumrad [71]	2018	Identify major areas of safety concern in oncology and to offer suggestions and actions for improvement to prevent similar events from reoccurring.	National Center for Patient Safety RCA database	2013-2017	Patients with cancer treated at VA hospitals	NA	United States	Other: Harmful event type	Number (%)	10.5% of events	There were 48 reported oncologic adverse events that occurred between 2013 and 2017, the majority of which were delays in care, followed by chemotherapy errors. Suicide composed 10.5% of the events, all stemming from a lack in coordination of care, education of both the staff and patient, and communication.
Kendal [34]	2012	Examine factors associated with suicide and accidental deaths among cancer patients	SEER	1973-2007	Patients with cancer	N/A	United States	Sex; Age; Race; Time; Other: Cancer site, marital status, cancer stage	Proportional hazards analysis, cumulative distribution function, risk ratio for death	Overall not reported	The major identifiable risk factor for suicide was male gender. Minor risk factors for suicide included metastases, advanced age, and respiratory as well as head and neck tumors.

Yu [35]	2020	Identify potential risk factors related to suicide in leukemia patients	SEER	1975-2017	Leukemia patients	US general population	United States	Age; Race; Time; Geography; Other: Leukemia type, treatment received, primary diseases, household income	SMR	SMR = 2.16, 95% CI: 1.85 – 2.47	Older, white, or male patients have a higher risk of suicide compared to the general US population
Klaassen [16]	2015	Use a population-based cohort to assess the incidence of suicide among patients with genitourinary malignancies compared with the general US population.	SEER	1988-2010	Patients with prostate, bladder, kidney, testis, and penile cancer	US general population	United States	Age; Race; Other: Marital status, cancer type, cancer grade, disease stage, treatment received	SMR, OR for factors associated with suicide	Overall not reported	Genitourinary malignancies pose a significant risk of suicide, with the patients at highest risk being those with bladder and kidney cancer. Patients with prostate cancer demonstrated an increased prevalence of suicide with increasing time from diagnosis, and patients with bladder cancer appeared to have the highest incidence of suicide within the first 5 years after diagnosis
Wang [72]	2022	Describe the distribution of causes of death in thyroid cancer patients	SEER	1975-2016	Thyroid cancer patients with an index thyroid malignancy	US general population	United States	Sex; Age; Race; Time; Other: Marital status, cancer stage at diagnosis, treatment received non-cancer and cancer death causes	SMR, cumulative mortality rate, annual percentage change	SMR for non-cancer death = 1.59, 95% CI: 1.56-1.62	Patients with thyroid carcinoma have a higher risk of non-cancer deaths than the cancer-free population

Su [39]	2022	Characterize the patterns and risk factors for deaths from suicides and cardiovascular events among patients with multiple primary cancers in the United States	SEER	1975-2016	Patients with single or multiple primary cancers, excluding patients diagnosed only by autopsy reports or death certificates and without complete follow-up information, including age at diagnosis, follow-up duration, or race	US general population	United States	Sex; Age; Race; Time; Geography; Other: Median house-hold income, cancer stage, surgery received, number of primary cancers, cause of death	SMR	SMR = 1.89, 95% CI: 1.76-2.02	Mortality rates from suicide and cardiovascular diseases among patients with multiple primary cancers were significantly higher than those of patients with single primary cancers. In addition, patients with asynchronous multiple primary cancers had a higher risk of suicide and cardiovascular death than those with synchronous multiple primary cancers
Osazuwa-Peters [20]	2018	Evaluate differences in suicide based on gender and HPV-relatedness in head- and neck cancer patients	SEER	1973-2014	Adult patients with squamous cell carcinoma of the head and neck	US general population	United States	Sex; Age; Race; Other: Marital status, cancer stage, number of primary tumors, HPV-relatedness	SMR	Overall not reported	Male HNC survivors had significantly greater risks of suicide than females.

Weiner [12]	2021	(1) Assess contemporary, population-based data for causes of death during prostate cancer survivorship in the United States, (2) relate these causes to time periods after the prostate cancer diagnosis and provide results stratified by tumor stage and patient characteristics, and (3) compare the risk of death for each cause with the risk in the general US population	SEER	2000-2016	Men diagnosed with prostate cancer as their first malignant neoplasm	US general population	United States	Sex; Age; Race; Geography; Other: Stage of disease, marital status, treatment modality, income	SMR	SMR = 0.95, 95% CI: 0.90 – 1.00	Among men diagnosed with local/regional disease, deaths from non-prostate cancer causes were 4-fold more frequent (83% vs 17%), but these men were overall less likely to die of most non-prostate cancer causes in comparison with the general population. Men with distant disease have an increased risk of death from non-prostate cancer causes, particularly suicide and cardiac death.
Mahdi [28]	2011	Characterize the suicide rate among women with gynecologic cancer in the US relative to suicide rates among women in the general population, and to identify patient, disease and treatment characteristics that are associated with particularly high suicide rates	SEER	1988-2007	Patients with gynecologic cancer (ovarian, uterine, cervical, vaginal)	Women in the US general population	United States	Age; Race; Time; Other: Cancer site, marital status, cancer stage, treatment received, time since diagnosis	SMR	SMR = 1.4, 95% CI: 1.2 – 1.7	Younger age at diagnosis, high grade disease, and absence of surgical intervention were associated with higher risk of suicide.

Alanee [73]	2012	Determine mortality rates due to suicide in testis cancer patients, and to identify patient and disease characteristics associated with increased risk of suicide	SEER	1995-2008	Patients with testis cancer	Standardized U.S. population in 2005 age 25-44	United States	Age; Race; Time; Other: Cancer subtype, marital status	SMR	SMR = 1.2 (95% CI: 1.1 – 2.1)	Risk of suicide is increased by 20% in patients diagnosed with testis cancer
Zhou [24]	2018	Estimate the trend of suicide rate and identify the high-risk group of NSCLC patients	SEER	1973-2013	Patients diagnosed with non-small cell lung cancer between 1973-2013	US general Population	United States	Sex; Age; Race; Time; Geography; Other: Marital status, site, grade, histology, stage, treatment received	SMR	Overall not reported	Male, unmarried, and elderly patients were at higher risk of suicide mortality
Turaga [74]	2011	Identify the suicide rate among patients with pancreatic cancer, using population-based data to identify patient, disease, and therapy characteristics associated with commission of suicide	SEER	1995-2005	Patients with pancreatic adenocarcinoma	US general population	United States	Sex; Age; Race; Time; Other: Marital status, disease stage, treatment received	SMR, cause-specific mortality for suicide	SMR = 10.8, 95% CI: 9.2 – 12.7	Male patients with pancreatic adenocarcinoma have a risk of suicide nearly 11 times that of the general population. Patients who undergo an operative intervention are more likely to commit suicide, generally in the early postoperative period

Yang [75]	2022	Examine the risk of suicide among cancer survivors who developed a second malignant neoplasm	SEER	1975-2016	Patients who developed a second malignant neoplasm during follow-up	US general population	United States	Sex; Age; Race; Time; Geography; Other: Cohabitation status, insurance, tumor size and grade, treatment modality, lifestyle factors, site of second malignant neoplasm	HR, SMR	HR = 1.23, 95% CI: 1.14 – 1.31	Compared with either patients with only a first malignant neoplasm or the general population, cancer survivors who received a second malignant neoplasm diagnosis were at increased risk of suicide death
Abdel-Rahman [40]	2019	Assess the socioeconomic predictors of suicide risk among cancer patients in the United States	SEER	2000-2010	Cancer patients diagnosed between 2000-2010	US general population	United States	Sex; Age; Race; Other: Socioeconomic Status, tumor site, SEER stage, marital status, ethnicity	Observed/Expected ratio for suicide among cancer patients	Overall not reported	Cancer patients in socioeconomically vulnerable environments have higher suicide risk than other cancer patients
Fang [76]	2009	Explore whether suicide and cardiovascular events are affected by a prostate cancer diagnosis	SEER, National Death Index	1979-2004	Patients with prostate cancer	US general male population	United States	Age; Race; Time; Geography; Other: Marital status, county-level education, county-level poverty, tumor grade, tumor stage	SMR	SMR during the first year after diagnosis = 1.4, 95% CI: 1.2 – 1.6	There was an increased risk of suicide and cardiovascular death among men who were newly diagnosed with prostate cancer. The excess risks were higher in periods closer to cancer diagnosis and in earlier calendar years.
Saad [77]	2020	Investigate the increase in suicide rate associated with diagnosis with brain cancer	SEER	2000-2016	Patients diagnosed with brain cancers	US general population	United States	Sex; Age; Race; Other: Marital status, tumor histology	Observed to expected event ratio	First year observed/expected = 3.05 (2.04 – 4.37)	Diagnosis with brain cancer is associated with an increased risk of suicide within the first year following diagnosis.

Sugawara [22]	2016	Examine the rate of suicide in patients with gastric cancer and to identify factors associated with increased risk of suicide	SEER	1998-2011	Patients diagnosed with gastric cancer, and who had one of the following histologic types: 'carcinoma, not otherwise specified (NOS)', 'adenocarcinoma NOS', 'adenocarcinoma intestinal type', 'carcinoma, diffuse type', 'tubular adenocarcinoma', 'adenocarcinoma with mixed subtypes', 'papillary adenocarcinoma, NOS', 'mixed cell adenocarcinoma', 'mucinous adenocarcinoma',	US general population	United States	Sex; Age; Race; Time; Other: Marital status, extent of disease, tumor grade and histology, treatment received	SMR, incidence rate ratios	SMR = 4.07, 95% CI: 3.18 – 5.13	Patients with gastric cancer have a higher risk of suicide than the general US population. The rate of suicide is highest within the first 3 months after diagnosis. Male sex, White race, unmarried status, and distant stage disease are significantly associated with increased risk of suicide.

					‘mucin-producing adenocarcinoma’, or ‘signet ring cell carcinoma’, excluding those first diagnosed at autopsy						
Siracuse [78]	2017	Determine (1) the incidence of suicide in patients with bone and soft tissue cancer, (2) whether the incidence of suicide is greater in patients with bone and soft tissue cancer than it is in the general US population, and (3) any demographic and tumor characteristics associated with increased suicide incidence	SEER	1973-2013	Patients with a primary bone or soft tissue tumor	National Center for Health Statistics of the US Census Bureau through SEER	United States	Sex; Age; Race; Time; Other: Marital status, stage at presentation, years since diagnosis, treatment received, cancer site	SMR	First 5 years since diagnosis SMR = 10.75, 95% CI: 9.19 – 12.61	The suicide incidence in the population with bone and soft tissue cancer was more than twice that of the general US population. Compared with subpopulations examined in other studies, the suicide incidence of patients with bone and soft tissue cancer ranked below the incidence of suicide in patients with cancer of the lung and bronchus, stomach, oral cavity and pharynx, larynx, and ovary. Higher suicide incidences were seen in men, patients of white race, patients who had cancer of the pelvis or vertebral column, and patients within the first 5 years of diagnosis.

Ma [36]	2021	Determine the suicide risk among patients with a primary solid tumor while identifying coincident patterns with high suicide risk according to the area-based socioeconomic status.	SEER	1975-2016	Patients with a primary solid tumor: brain, breast, colorectum, esophagus, kidney, larynx, liver, lung/bronchus, melanoma/skin, oral cavity, pancreas, prostate, small intestine, stomach, testis, thyroid, urinary bladder	N/A	United States	Sex; Age; Race; Other: Cancer stage, grade, insurance status, marital status, community-level socioeconomic status	Cox-proportional hazards model	Overall not reported	The risk of suicide death was significantly higher among adult patients with a primary solid tumor who were older, male, white, had not received active treatment, had no insurance, had high levels of cancer severity, and were unmarried.
Chen [37]	2020	Calculate the suicide rate and standardized mortality ratios and potential risk factors associated with suicide among hepatocellular carcinoma patients.	SEER	1975-2016	Patients with hepatocellular carcinoma	US general Population	United States	Sex; Age; Race; Time; Geography; Other: Marital status, Cancer stage, cancer treatments received	Suicide rate (# observed per 100,000 person years), SMR	SMR = 2.26, 95% CI: 1.78 – 2.84	Suicide rate among hepatocellular carcinoma patients was higher than in the general population. Earlier year of diagnosis, male sex, older age of diagnosis, white race were predictors of suicide.

Horn [79]	2020	Assess the risk of death from a primary cancer diagnosis versus competing causes of death and characterize the relative risk of death after a pediatric cancer diagnosis versus the general population	SEER	1980-2015	Pediatric patients diagnosed with cancer	US general population	United States	Sex; Age; Race; Time; Other: Cause of death	Risk of death by cause, SMR	Overall not reported	The risk of mortality for pediatric patients with cancer was significantly higher than the risk for the general population and certain noncancerous causes of mortality presented a particularly increased risk. The SMRs for all causes of death were generally highest within the first 5 years after diagnosis, although the risks remained significantly increased up to 30 years after the initial diagnosis, in particular for cardiovascular disease, suicide, infections, and accidents
Chen [10]	2021	Measure suicide rates and standardized mortality rates among esophageal cancer patients in comparison with the United States general population and recognize underlying factors associated with suicide	SEER	1975-2016	Esophageal cancer patients	US general population	United States	Sex; Age; Race; Time; Other: Primary site (within esophagus), histologic grade, marital status, SEER disease stage, treatments received, survival months post-diagnosis	SMR	SMR = 5.45, 95% CI: 4.66 – 6.35	Suicide rates among patients with esophageal cancer were greater than in the general U.S. population, especially in men, older people, and patients not receiving chemotherapy or surgery.

Yu [30]	2012	Study non–cancer-related mortality rates over time and examine the possible causes for several major deaths in patients with oral cavity and oropharyngeal cancer	SEER	1973-2007	Patients with primary malignant oral cavity and oropharyngeal cancer	N/A	United States	Sex; Age; Race; Time; Other: Marital status, tumor stage, treatment received, tumor site, ethnicity	SMR, risk ratios	SMR = 7.8, 95% CI: 4.6 – 12.4	Suicide rates among patients with oral cavity and oropharyngeal cancer have significantly increased over the past 3 decades.
Gaitanidis [26]	2017	Identify the trends in the incidence of suicide mortality among breast cancer in the United States over the past decades and associated risk factors	SEER	1973-2013	Patients with a breast cancer diagnosis who died of any cause	N/A	United States	Sex; Age; Race; Time; Geography; Other: Marital status, treatment received, cancer grade, cancer stage, cancer type, time since diagnosis	Chi-Squared, Multivariable logistic regression	Overall not reported	Younger age, male sex, nonwhite-nonblack race, single or separated/divorced/widowed marital status, cancer-directed surgery, lower tumor grade, earlier disease stage, progesterone receptor positivity, and shorter period elapsed from diagnosis were independent risk factors for committing suicide.
Shen [80]	2020	To define the suicide rate and explore potential risk factors related to suicide in patients with Kaposi's Sarcoma by using SEER database	SEER	1980-2016	Patients with Kaposi's Sarcoma older than 18 years from the SEER database (1980–2016)	US general population.	United States	Sex; Age; Race; Other: Year of diagnosis Marital status Tumor risk classification surgery (yes, no, unknown) radiotherapy (yes, no, unknown) chemotherapy (yes, no/unknown)	SMR	SMR = 7.64, 95% CI: 6.28 – 9.21	There is a higher suicide rate in Kaposi's sarcoma patients than the general population. Black race, advanced age of diagnosis, and chemotherapy were protective factors for suicide among Kaposi's sarcoma patients.

Ma [9]	2022	Determine the suicide rate of pancreatic cancer patients from 2000 to 2018 in the U.S., compared with the general population in different demographic and clinicopathological subgroups, identify factors that potentially increase suicide risk, and provide research evidence for reducing the risk of suicide in patients with pancreatic cancer.	SEER	2000-2018	Patients with pancreatic cancer	US general population	United States	Sex; Age; Race; Time; Other: Cancer type, cancer stage, treatment received	Suicide rates (# observed per 100,000 person-years), SMR	SMR = 6.43, 95% CI: 5.49 – 7.37	Patients with pancreatic cancer had an elevated risk of suicide compared to the general US population. Male sex, white race, unmarried status, and diagnosis of pancreatic adenocarcinoma was associated with higher risk of suicide, while cancer-directed surgery and chemotherapy were associated with lower risk.
Aboumrad [71]	2018	Gain a better understanding of health system factors that may contribute to suicide in patients with cancer and identify potential strategies for care teams to address this using root cause analysis method for adverse events	National Center for Patient Safety Root Cause Analysis database	2002-2017	Patients with cancer treated in the Veterans Health Administration	N/A	United States	Sex; Age; Other: stage in care, cancer type, pre-existing mental health problems, health complications, stressors, time since last medical visit, location of last visit, type of last visit, mental health referral status, method of suicide	Root cause of suicide	Overall not reported	The majority of suicides occurred within 7 days since the last medical visit, the main primary cancer types were prostate and lung, the main suicide method was firearm, suicides happened during palliative care or upon diagnosis, most patients were not referred to mental health services

Barnes [81]	2022	Assess whether individuals with a childhood cancer diagnosis are at increased risk for suicide with an emphasis on potential changes in suicide risk by attained age, and examine associations between sociodemographic and cancer diagnosis risk factors and the risk of suicide	SEER	1975-2016	Individuals diagnosed with a first primary malignancy at the ages of 0 to 19 years	US general population	United States	Sex; Age; Race; Time; Geography; Other: Ethnicity, cancer site, stage at diagnosis	SMR	Overall SMR = 1.14, 95% CI: 0.91 – 1.43. Beyond 28 years: SMR = 1.40, 95% CI: 1.02 – 1.87	There may be a significantly elevated risk of suicide for long-term survivors of a childhood cancer diagnosis
Guo [82]	2019	Characterize the suicide rates and SMRs among kidney cancer patients and identify the potential risk factors relevant to suicide based on the SEER database	SEER	1973-2015	People diagnosed with kidney cancer	US general population	United States	Sex; Age; Race; Time; Other: Marital status, cancer stage, cancer subtype, treatment received	SMR	SMR = 1.83, 95% CI: 1.59 – 2.10	Patients with kidney cancer had an increased risk of suicide compared to the general US population. Earlier year of diagnosis, black race, higher histologic grade, and cancer-directed surgery not performed were significant predictors of suicide.

Urban [25]	2013	Explore whether demographic, disease, and treatment variables can be used to identify subgroups of patients with lung cancer with differing risks of suicide in a large, US cohort of patients with lung cancer	SEER	1973-2009	All patients with cancer with malignant behavior of the lung and bronchus, excluding cases where the age was not known or the histology was not compatible with either a lung cancer primary or mesothelioma	US general population	United States	Sex; Age; Race; Time; Geography; Other: Treatment received, poverty and education levels, marital status, primary tumors (single vs. multiple), cancer stage, type of lung cancer	SMR	SMR = 4.95, 95% CI: 4.68 – 5.24	All patients with lung cancer have an excess risk of suicide immediately after diagnosis. However, while those with poorer prognoses have a greater risk.
Guo [83]	2022	Establish an association between causes of death and demographics in prostate cancer and compare risk of causes of death with the general US population	SEER	2000-2016	Patients with prostate cancer	US general population	United States	Age; Race; Time; Other: Cancer stage and grade, treatment received, survival time	SMR	SMR = 0:97, 95% CI: 0:92 − 1:02	The risk of death from non-cancer causes within 5 years of prostate cancer diagnosis was significantly higher for infectious diseases, septicemia, and suicide. However, the overall risk of suicide death was not different among prostate cancer patients as compared to the general US population

Saad [77]	2019	Assess the risk of suicide within the first year after a cancer diagnosis, to determine whether the incidence of suicide in patients with cancer is greater than the incidence in the normal population in the United States, and to determine whether there is any association between demographics or tumor characteristics and suicidal incidence	SEER	2000-2014	Patients with cancer	US general population	United States	Sex; Age; Race; Other: Marital status, stage, cancer site, cancer type	Observed/expected ratio for suicide	O/E = 2.52, 95% CI: 2.39 – 2.64	The risk of suicide increases significantly within the first year after a diagnosis of cancer in comparison with the general population. The risk of suicide is specifically higher after a diagnosis of pancreatic, lung, or colorectal cancer, whereas a diagnosis of breast or prostate cancer does not result in an increased suicide risk.
Anderson [84]	2018	Investigate suicide rates among patients with cancers of the digestive system compared to the general population and examine whether suicide rates are higher in subgroups by site, stage, time since diagnose, patient characteristics	SEER	2000-2014	Patients with digestive system tumors: esophagus, colon, rectum, liver, pancreas, stomach, other	U.S. general population	United States	Sex; Age; Race; Other: Cancer site, marital status, Stage, Surgical treatment	SMR	SMR=1.91, 95% CI: 1.79 - 2.04	All cancer sites had significantly higher suicide rates than the general population. The highest standardized mortality ratios were for esophageal, pancreatic, stomach, and liver cancers.

Yang [85]	2019	To identify the incidence of and risk factors for suicide death in male genital-system cancer patients in the United States.	SEER	2004-2015	Male genital-system cancer (MSGC) patients older than 18, excluding cases without a diagnosis, or microscopic confirmation, only with autopsy findings, or incomplete variables	N/A	United States	Sex; Age; Race; Time; Other: Marital status, primary site, cancer stage, treatment received, insurance status	Univariate and multivariate logistic regression models	Overall not reported	MGSC patients of younger age, being unmarried, being DSW (divorced, separated, or widowed), and no/unknown surgery significantly increased the risk of suicide death. Importantly, a time of<1 year after the diagnosis was related to an increased risk of suicide death
Chen [86]	2022	Determine the relative risk of suicide and accidental deaths among ovarian cancer patients compared with the general population and to identify demographic and tumor-related characteristics that are associated with a particularly high risk of suicide and accidental deaths among ovarian cancer patients.	SEER	1973-2016	Patients with primary ovarian cancer	US general population	United States	Age; Race; Time; Geography; Other: Education, income, tumor grade, stage, treatment received	Relative risk for death by suicide or accidental death	SMR = 1.86, 95% CI: 1.54 – 2.25	Risk of suicide and accidental death was higher among women with ovarian cancers compared with the general US population

Rahouma [31]	2018	Analyze suicide rates among cancer patients in the United States relative to the general US population, aiming to classify types of cancers according to the risk of suicide, in order to help prioritize the implementation of survivorship plans and to guide discussion and counselling of patients.	SEER	2018	Cancer patients diagnosed between 1973 and 2013	US general population	United States	Sex; Age; Race	SMR	SMR = 1.57, 95% CI: 1.53 – 1.61	The highest suicide risk was observed in patients with lung cancer, followed by colorectal, breast, and prostate cancer.
Samawi [87]	2017	Examine the incidence and predictors of suicide for patients with rectal cancer and with colon cancer.	SEER	1973-2009	Adult patients with confirmed adenocarcinoma of the colon or rectum during 1973–2009		United States	Sex; Age; Race; Other: marital status (single, married, or unknown status primary tumour site (colon vs. rectum), stage at diagnosis (local or regional vs. distant), number of primary tumours, whether surgery to the primary site was performed.	Kaplan–Meier survival analysis, multivariate adjusted Cox proportional hazards regression were used	HR = 1.05, 95% CI: 0.83 – 1.33	The suicide risk in CRC patients is low (<0.2%), and no difference was found based on location of the primary tumor. Sex, age, distance spread of disease, and intact primary tumor were the main predictors of suicide among CRC patients.

Zhang [88]	2021	Develop and validate a risk assessment model to predict the probability of suicide within 5 years after the diagnosis of lung cancer	SEER	2007-2016	Lung cancer patients diagnosed between 2007 to 2016	US general population	United States	Sex; Age; Other: marital status, grade, histology, stage, treatment received	Nomogram, risk matrix	C-index = 0.77	Demographic information such as sex and marital status, and clinical factors including tumour grade and treatment history of surgery, radiation and chemotherapy were important predictors of suicide risk.
Yu [89]	2021	Determine whether patients with primary malignant bone tumors are at a higher risk of suicide and accidental death than the general population and identify the demographic and tumor-related characteristics and type of surgery associated with a higher risk of suicide and accidental death among these patients	SEER	1973-1975	Patients with primary malignant bone tumors diagnosed between 1973 to 1975	US general population	United States	Sex; Age; Race; Time; Other: Primary site, clinical stage	SMR	SMR = 2.17, 95% CI: 1.80-2.62	Patients with primary malignant bone tumors had a higher risk of suicide and accidental death than the general population in the United States
Osazuwa-Peters [90]	2021	To evaluate whether the risk of suicide among patients with HNC differs by rural vs. urban or metropolitan residence status	SEER	2000-2016	Patients with head-and-neck cancer	US general population	United States	Sex; Age; Race; Geography; Other: Cancer site, marital status, county-level income, county-level education level, cancer stage	SMR	Overall not reported	Suicide incidence is elevated for patients with head-and-neck cancer, particularly those living in rural areas

Dalela [91]	2015	Delineate the demographic and tumor-related factors that impact the proclivity of suicide and accidental death in American men with prostate cancer	SEER	1988-2010	Men diagnosed with adenocarcinoma of the prostate	Men with other solid cancers	United States	Age; Race; Time; Geography; Other: Time to suicide since diagnosis, type of primary treatment, definitive primary treatment, stage at diagnosis, marital status, county-level educational attainment, county-level family income	Risk of suicidal and accidental death	ARR = 3.98, 95% CI: 3.02 – 5.23	Men diagnosed with prostate cancer have a significantly higher risk of suicidal and accidental death within the first year of diagnosis than their counterparts diagnosed with other solid cancers.
Barnes [81]	2022	Determine whether adoption of the Affordable Care Act was associated with suicide incidence in patients with cancer in the USA and isolate the association between state Medicaid expansions and suicide incidence.	SEER	2011-2016	Patients diagnosed with cancer	18-64 year old patients vs. 65-74 year old patients, patients in Medicaid expansion vs. non-expansion states	United States	Sex; Age; Race; Other: Residence (metropolitan vs. non-metropolitan), County-level education, County-level income,	Cumulative suicide incidence within 1 year of cancer diagnosis	Pre-ACA: Adjusted change = -6.14, 95% CI: -13.48 – 1.19	There was no significant change in the incidence of suicide for 18–64-year-old patients with cancer after 2014, even in Medicaid expansion states. However, there was a significant decrease in suicide incidence after 2014 among 55–64-year-old patients with cancer.

Kam [18]	2015	Examine suicide rates among patients with cancers of the head and neck	SEER	1973-2011	Patients with head and neck cancer: nasal cavity, nasal sinuses, nasopharynx, oral cavity, oropharynx, salivary glands, hypopharynx, larynx, thyroid gland	US general population	United States	Sex; Race; Time; Other: Treatment received, cancer stage, cancer site	Suicide rate, SMR	SMR = 3.21, 95% CI: 2.18 – 4.23	Suicide rates among patients with head and neck cancer are significantly higher than that of the general population. In particular, patients with hypopharyngeal and laryngeal cancer are at highest risk
Han [29]	2021	Examine trends in cancer-related suicides in comparison to trends in overall suicides and by high-risk subpopulations, including older, male, and rural residents	CDC WON-DER	1999-2018	Cancer patients	Overall suicide rate	United States	Sex; Age; Race; Time; Geography; Other: Co-contributing causes (mental health, heart disease)	Age-adjusted cancer-related suicide rate	AAPC = -2.8 %, 95% CI: -3.5%– 2.1%	There was a decreasing trend of cancer-related suicide, in contrast to an increasing rate of overall suicides. The largest declines were among high-risk populations

Suk [8]	2021	To examine whether the risks and patterns of suicide mortality among people with a cancer diagnosis differ by US county-level median income and rural or urban status	SEER	2000-2016	People diagnosed with a first primary malignant tumor, excluding those diagnosed from death certificates or autopsy and if ages at the times of diagnosis were unknown	Mortality data from the US National Center for Health Statistics	United States	Sex; Age; Race; Time; Geography; Other: Ethnicity, county-level rural and urban status, county-level income, educational level attainment, unemployment rate, and percentage of black residents	SMR	SMR = 1.41, 95% CI: 1.38 – 1.44	There is higher suicide mortality following cancer diagnosis among people living in lower-income and rural counties.
Osazuwa-Peters [20]	2018	Examine mortality rates from suicide among survivors of head-and-and neck cancer and among survivors of cancer of other common sites, and to compare the risk of suicide among head-and-neck cancer survivors with the risk among survivors of other cancers	SEER	2000-2014	Patients with a first primary malignancy of the head and neck	Survivors of other common cancers besides head-and-neck cancer	United States	Sex; Age; Race; Other: Cancer site, marital status, cancer stage	Adjusted Risk Ratio	ARR = 1.97, 95% CI: 1.77 – 2.19	Suicide risk was elevated in survivors of head-and-neck cancer compared with survivors of other major cancers
Zhou [50]	2019	Analyze the necessity to separate adolescent and young adult cancer patients as a special group to identify high risk patients	SEER	1973-2015	Patients with solid malignancies	US general population	United States	Sex; Age; Race; Time; Other: Cancer type, marital status, tumor stage, treatment received	SMR	Overall not reported	Male sex, white race, unmarried status, earlier stage and later time from cancer diagnosis are suicide risk factors for both AYA and all-age cancer patients.

Jayakrishnan [92]	2015	Understand the effect of surgical morbidity on risk of suicide among patients with solid tumors and to examine the impact of cancer prognosis on this interaction.	SEER	2004-2011	Patients diagnosed with stage II-III cancer undergoing curative surgery	US general population	United States	Sex; Age; Race; Other: Cancer type, surgical morbidity, cancer prognosis, marital status	Incidence rates, odds ratios, and hazard ratios for suicide	Incidence rate = 16.58, 95% CI: 14.54 – 18.82	Patients undergoing surgeries of high morbidity are more likely to commit suicide even after adjusting for the prognosis of disease. Single, elderly men undergoing such surgeries are particularly vulnerable.
Heynemann [93]	2021	Investigate clinical and demographic factors associated with death by suicide among adolescents and young adults with cancer compared to US population normative data and other adolescents and young adult individuals with cancer	SEER	1975-2016	Adolescents and young adults (15-39 years) with cancer	US general population	United States	Sex; Age; Race; Time; Other: Cancer site, treatment received, cancer grade, cancer stage, number of primary tumors, relationship status	SMR, odds ratios	SMR = 34.1, 95% CI: 31.4–36.9.	Adolescents and young adults with cancer were 34 times more likely than the general population to have died by suicide.
Yang [94]	2021	Characterize suicide mortality among adolescent and young adult (AYA) patients in the US and identify risk factors associated with a higher risk of suicide	SEER	1973-2015	AYAs and older adults with cancer	US general AYA population	United States	Sex; Age; Race; Time; Other: Marital status, stage of cancer, cancer site, cause of death, number of primary tumors	SMR	SMR = 1.234, 95% CI: 1.159 – 1.313	The incidence of suicide in AYA patients with cancer in the US was approximately 23.4% higher than that of the general US population

Pham [95]	2018	Assess incidence of suicide in patients with colon and rectal malignancies, while expounding upon demographical, clinical, and diagnostic factors associated with suicide in this patient population	SEER	1988-2010	Patients diagnosed with colon and rectal malignancies	WISQARS	United States	Sex; Age; Race; Other: Income, marital status, and clinical variables	Contingency tables, mortality ratios, and odds ratios	OR = 1.53, 95% CI: 1.13 – 1.33	Race and gender appear to influence suicide rates in patients with colorectal cancer. Females with colorectal cancer demonstrated approximately four times the suicide rate of the gender-matched population. Also, the research indicates that suicide incidence is a factor in this diagnosis without preference for stage or sub-type. The increased suicide risk in patients with CRC does not seem to be associated with poor prognosis or likelihood of mortality.
Fu [96]	2021	Examine the suicide rates among childhood cancer survivors in the US and to determine characteristics that are associated with higher suicide risk, as well as the trend of suicide rates over age and years after diagnosis	SEER	1975-2016	Survivors of pediatric cancer	N/A	United States	Sex; Age; Race; Time; Other: Median household income, Normalized cost-of-living index, Cancer site, Surgery received (yes or no)	Frequency of suicide death	Overall not reported	Most suicides occurred after the age of 15. Suicide was particularly a problem for survivors with thyroid cancer.

Bowden [21]	2017	Examine suicide incidence and associated factors in gastric cancer patients	SEER	1973-2013	Gastric cancer patients	US general population	United States	Sex; Age; Race; Other: Time since diagnosis	SMR	SMR = 3.21, 95% CI: 2.80-3.67	Patients with gastric cancer have a higher rate of suicide compared to the general U.S. population. Female gender, White race, age ≤39 years, and age 70–79 years are factors associated with increased risk of suicide in patients with gastric cancer.
Simpson [97]	2018	Investigate the risk of suicide in patients with penile cancer	SEER	1973-2013	Patients with penile cancer	US general population	United States	Age; Race; Other: Marital status, tumor grade, treatment received	SMR, suicide rates (number of suicides/person-years of survival)	SMR = 1.58, 95% CI: 1.18-2.11	Penile cancer does increase the risk for suicide compared with age-adjusted controls
Du [38]	2021	Examine the association between insurance status and risk of suicide mortality in cancer patients	SEER	2007-2016	Patients with cancer	Insured vs. Any Medicaid vs. Uninsured	United States	Sex; Age; Race; Time; Other: Cancer type, county-level income, county-level unemployment rate, county-level education, cancer stage, marital status	Risk of suicide death	Overall not reported	Uninsured patients with cancer have a greater risk of suicide mortality than insured patients

Elshanbary [23]	2021	Identify the risk factors for suicide in patients with gastric adenocarcinoma	SEER	1975-2016	Patients with gastric adenocarcinoma	US general population	United States	Sex; Age; Race; Other: Marital status, primary cancer site, treatment received, cancer stage, insurance status	SMR	Overall not reported	The risk of suicide mortality was higher among gastric adenocarcinoma patients than in the general US population. The risk of suicide was lower in female and Black patients
Zaorsky [98]	2016	Characterize the causes of death among cancer patients in the United States as a function of calendar year, patient age, and time after diagnosis.	SEER	1973-2012	Cancer patients diagnosed between 1973 to 2012	US general population	United States	Age; Time; Other: Cancer type	SMR	Overall not reported	Among the patients dying from suicide and self-inflicted injury, those with lung and bronchus cancer had the highest Standardized Mortality Ratio within the first year, followed by breast cancer and testis cancer. Testis cancer patients, however, had the highest persistent risk of suicide and self-inflicted injury.
Zaorsky [4]	2019	Identify cancer patients at the highest risk of suicide compared to the general population and other cancer patients.	SEER	1973-2014	Cancer patients diagnosed between 1973-2014	US general population	United States	Sex; Age; Race; Time; Other: Marital status, cancer stage, surgery status	SMR	SMR = 4.44, 95% CI: 4.33 – 4.55	Elderly, white, unmarried males with localized disease are at highest risk vs other cancer patients. Among those diagnosed at < 50 years of age, the plurality of suicides are from hematologic and testicular tumors; if > 50, from prostate, lung, and colorectal cancer patients.
